# Event and Comment

**Published:** 1909-09-15

**Authors:** 


### DENTAL REGISTER.

**Vol. LXIII. September 15, 1909. No. 9.**

### EVENT AND COMMENT.

PLETHORIC PROGRAMMES: In this day of big things and extraordinary efforts to claim the notice of the public by excessive demonstrations, it would seem that the dignified spirit which has always been an idolized ideal is threatened by the strenuous efforts made by our zealous committeemen who make the programmes for our professional societies. The recent meeting of the American Medical Association offers an excuse for this comment. The twelve sections presenting the programme for that meeting listed 353 papers and addresses, to be delivered and discussed within four days time, and in five sessions of an average of three hours each. Of course they were not all delivered in one place, but even if the twelve sections were all deliberating separately and simultaneous, an average of nearly six papers would have to be read and discussed at every one of the twelve sections at each session. This would mean on the average about 30 minutes for the consideration of each paper. Is it possible for any body of men to read and thoroughly consider 23 papers on as many important subjects in 15 hours even though the time be spread over four days of time? It is a waste of energy and no great amount of knowledge, skill or culture can be disseminated by any such cramming process. Besides it tends strongly in the direction of the boasting habit which all good professional people condemn as undignified and unethical. We should hesitate to announce our doings in such a way as to give out the idea that it is professional to demonstrate in so large a style even for the sake of attracting the unwilling and inactive members of the profession. We have many times questioned the idea that seems to prevail that we must have large attendances at our meetings, and to get them that we must advertise in some sensational manner. There can be no question but attendance on professional gatherings is a habit that some men can't shake off, and which others seem never to have acquired. The point however, that we wish to make is that no thoroughness and little of value in the way of permanence can result from the present notion we have that the meeting must be big in numbers, and to secure such attendance the programme must be long and exhausting. In our judgement one good paper well digested at each session will accomplish more in the way of real culture than five or six papers presented in the same period to the great weariness and disgust of most people who hear them. The management of the section on Stomatology of the American Medical Association is endeavoring to impress the medical profession with the kind of work being done by the dental profession, and possibly, it may be succeeding, as all the papers are printed in the Journal of the Association, and for 23 weeks this coming year 50,000 medical practitioners will have a chance to read a paper on some subject of dental concern, and of related interest to the medical practitioner. If this can be accomplished with any reasonable proportion of the members, it will unquestionably mean much to us. How far the medical men read such articles, of course, we do not know, and for this reason it may perhaps be justifiable to utilize this opportunity to accomplish a better understanding of our aims by the general medical practitioner. The congested programme is not confined entirely to the section of Stomatology, however but seems equally prevalent in many of our dental meetings.

